# Experiences and attitudes of early career psychiatrists towards ECT – an international study

**DOI:** 10.1192/j.eurpsy.2024.1462

**Published:** 2024-08-27

**Authors:** C. Tapoi, C. Noël, R. de Filippis, D. Gurrea Salas, K. Mieze, D. Almeida, A. Pushko, A. Wilkowska, M. E. Gołębiewska, L. Alexander, M. Pinto da Costa

**Affiliations:** ^1^Department of Psychiatry, Prof. Dr. Dimitrie Gerota Emergency Hospital, Bucharest, Romania; ^2^Department of Psychiatry, Centre Hospitalier Universitaire Saint-Pierre, Université Libre de Bruxelles, Bruxelles; ^3^Child and Adolescent Psychiatry Hospital La Petite Maison, Chastre, Belgium; ^4^Department of Health Sciences, University Magna Graecia of Catanzaro, Catanzaro, Italy; ^5^Department of Addictive Disorders, Psychiatric Services Aargau, Brugg, Switzerland; ^6^Department of Doctoral Studies, Riga Stradins University, Riga, Latvia; ^7^Department of Psychiatry and Mental Health, Hospital de Loures, Loures, Portugal; ^8^National Medical University, Communal Non-Commercial Enterprise Precarpathian Regional Clinical Center for Mental Health of the Ivano-Frankivsk Regional Council, Ivano-Frankivsk , Ukraine; ^9^Department of Psychiatry; ^10^Department of Developmental, Psychotic, and Geriatric Psychiatry, Medical University of Gdańsk, Gdańsk, Poland; ^11^Institute of Psychiatry, Psychology & Neuroscience, King’s College London, London, United Kingdom; ^12^Institute of Biomedical Sciences Abel Salazar, University of Porto, Porto, Portugal

## Abstract

**Introduction:**

Electroconvulsive therapy (ECT) is a psychiatric intervention that has proven effectiveness and safety in various psychiatric conditions, such as major depressive disorder, prolonged or severe manic episodes and catatonia. Despite positive scientific evidence, ECT was always seen as controversial by patients, caregivers, and even some psychiatrists, which lead to a decrease in its use over the years.

**Objectives:**

To investigate the way young psychiatrists view the place of ECT in modern psychiatry by assessing their knowledge, attitude and access to training opportunities in ECT.

**Methods:**

An anonymous survey was disseminated online among early career psychiatrists and psychiatric trainees. The questionnaire consisted of 36 multiple-choice and Likert scale questions.

**Results:**

Most of our respondents consider ECT both an effective and a safe treatment option and would recommend ECT to their patients when indicated. Early career psychiatrists who had access to ECT training are more knowledgeable about the indications, precautions and side effects of this method, but more than half of the participants mentioned ECT training was unavailable during their residency programme. Almost all respondents stated that they are interested in enhancing their theoretical and practical competencies in ECT.

**Conclusions:**

Early career psychiatrists have a positive attitude towards ECT but express the need of targeted education aimed at improving levels of knowledge about ECT.

**Disclosure of Interest:**

None Declared

